# Knowledge and Attitude of Saudi Students towards Plagiarism—A Cross-Sectional Survey Study

**DOI:** 10.3390/ijerph182312303

**Published:** 2021-11-23

**Authors:** Rakhi Issrani, Abdulrahman Alduraywish, Namdeo Prabhu, Mohammad Khursheed Alam, Rehana Basri, Fahad Muqbil Aljohani, Mshari Ali Abdullah Alolait, Alaa Yahya Ali Alghamdi, Modhi Mohammed Nasser Alfawzan, Abdullah Hamdan Mashog Alruwili

**Affiliations:** 1 Department of Preventive Dentistry, College of Dentistry, Jouf University, Sakaka 72341, Saudi Arabia; mkalam@ju.edu.sa; 2 Department of Internal Medicine, College of Medicine, Jouf University, Sakaka 72341, Saudi Arabia; dr-aaad@ju.edu.sa; 3 Department of Oral & Maxillofacial Surgery and Diagnostic Sciences, College of Dentistry, Jouf University, Sakaka 72341, Saudi Arabia; ntprabhu@ju.edu.sa; 4 Neurology Division, Department of Internal Medicine, College of Medicine, Jouf University, Sakaka 72341, Saudi Arabia; drlamisha@gmail.com; 5 Aja Primary Health Care Centre, Ministry of Health, Hail 55471, Saudi Arabia; faljohani19@moh.gov.sa (F.M.A.); m.m.alfozan@hotmail.com (M.M.N.A.); 6 Safad Dental Center, Riyadh 11451, Saudi Arabia; drmshari90@gmail.com; 7 Badanh Primary Health Care Centre, Arar 91431, Saudi Arabia; alaa.y.g@hotmail.com; 8 Ministry of Health, Sakaka 42421, Saudi Arabia; dr.a.alrwaily@gmail.com

**Keywords:** attitude, knowledge, plagiarism, policy, questionnaire

## Abstract

Background: Plagiarism (Plg) is an unacceptable practice since it affects the integrity of scientific literature. Aim and objectives: To seek students’ knowledge and attitude regarding Plg and solicit suggestions to prevent Plg in our institute. The specific objectives of the study were to ascertain whether students’ knowledge and attitudes about Plg differ by their demographic characteristics and academic years. Methodology: A 32 item questionnaire was given to all the undergraduate (UG) students of the College of Medicine and Dentistry, Jouf University, KSA. The research questions focused on demographic information, knowledge and attitude regarding Plg, and suggestions to curb Plg. Results: A total of 134 UG students filled out the survey. The samples consisted of 97 males and 37 females. Most of the students displayed adequate knowledge regarding Plg in terms of copying words or ideas, quoting references, and copying words without changing the matter. As compared to female students, male students had better awareness regarding meaningful and harmful effects of practicing Plg (*p* < 0.05). Additionally, our results indicate that as students climb the academic ladder, their awareness on Plg tends to rise. Common reasons for plagiarizing are discussed here together with suggestions to combat Plg. Conclusion: The results of this study can be mainly taken as an eye opener which demonstrates the vital factors pertaining to the level of students’ knowledge about Plg, and to eradicate the problem, there is a need for more discussions and training on this topic for students.

## 1. Introduction

The name Plg is derived from the Latin word “plagiarius” meaning “kidnapping” or “plunderer” [1]. It refers to the use of other people’s ideas or words and includes the use of published or unpublished data in the original language or as a translation without acknowledging the author(s) [2]. In the field of science and in academic environment, Plg is considered a tremendous academic and an ethical breach as it constitutes theft of intellectual property [3]. Plg is neither a new thing nor can it be said to be a problem particular to Saudi universities; rather, it is a main reason for concern to the academic community all over the world [4]. In the last two decades, Plg has attained greater public attention due to the remarkable rise of the Internet and the associated ‘copy-paste culture’ of generation Z students [5].

Different studies show that many students, being the least experienced among researchers, commit Plg either unknowingly or due to lack of skills. The other reasons for students to indulge in Plg includes a vicious competitive academic environment, lack of knowledge about Plg, students’ propensity towards such behavior, poor time management, not realizing the seriousness of such violations, and problems with using foreign language [6]. For these reasons, the students are included as the target population and have been studied by various researchers in different countries but there is relatively less research in this area in Saudi Arabia.

Researches on topics such as Plg are of utmost importance to govern the prevalence of Plg. According to the “theory of planned behavior”, certain attitudes and subjective norms are responsible for behavior change [7]. Therefore, there is an urgent need of educational modifications and policies to combat Plg at both the university and college level.

Against this backdrop, the present study was conducted with an aim to investigate students’ views on Plg, the degree to which they are knowledgeable about Plg, and the reasons leading them to plagiarize, if any. An additional objective was to ascertain whether students’ knowledge and attitude about Plg differ by their demographic characteristics and academic years. To reach these aims, the research questions of the study are:

1. To what extent are the students knowledgeable about Plg?
2. What is the students’ attitude towards Plg and how they perceive it?
3. What are the strategies to curb/prevent Plg?

## 2. Materials and Methods

*Study design:* A cross-sectional survey study.

*Study duration:* November 2019 to December 2019.

Ethical clearance (approval no. LCBE 09-03/41) was received from the Institutional Review Board and all the procedures in this study were in compliance with the Helsinki Declaration. 

*Sample population, size and characteristics:* Sample size estimation was performed for “Estimating Single Proportion with Finite population correction”. Anticipating a 40% prevalence of knowledge and attitude about Plg, an absolute precision of 5%, and a 95% confidence interval, the target population size of 200 and a sample size of 130 was found to be sufficient. 

Systematic random sampling (first subjects were selected through lottery method) was used for participant selection. For this study, a thirty-two item questionnaire (in Arabic and English Language as well) was conveniently hand delivered to the undergraduate students (4th and 5th academic year) and Interns of College of Medicine and Dentistry, Jouf University, KSA once regularly scheduled classes ended. Participation was made voluntary and the purpose of study was briefed to them before receiving verbal informed consent. It was assured in the beginning of the survey itself that the results of the questionnaire would be only presented/published as aggregate data, maintaining the confidentiality of personal information. The sample was further stratified based on gender, area of study, and academic year.

### 2.1. Research Questions

A questionnaire was crafted by following previous studies [3,4,8] to collect information regarding knowledge and attitude towards Plg. The overall questionnaire consisted of thirty-two closed-ended questions with variable options that were divided into four sections as follows:

(1) Section I consists of background information such as age, gender, academic year, etc.—Questions 1–6;
(2) Section II comprises knowledge factors of Plg measured on a five-point Likert scale anchored by “strongly agree” (1) to “strongly disagree” (5)—Questions 7–12;
(3) Section III constitutes students’ attitude towards Plg measured on a five-point Likert scale anchored by “strongly agree” (1) to “strongly disagree” (5)—Questions 13–27;
(4) Section IV comprises questions used to find out students’ attitudes to strategies for curbing Plg—Questions 28–32.

Reliability analysis demonstrated the Cronbach’s Alpha coefficients for this questionnaire ranged from 0.953 to 0.945 for different sections, which was considered as being relatively high and internally consistent.

### 2.2. Statistical Analysis

Data were analyzed using Statistical Package for Social Sciences (SPSS) version 21. Responses to all the items in the questionnaire were summarized as mean and standard deviation. Gender-wise and professional course-wise comparisons of mean score of knowledge and attitude towards different items of the questionnaire were performed using the Mann Whitney U test. Academic year-wise comparison was performed using the Kruskal Wallis test. The level of statistical significance was set at *p* ≤ 0.05.

## 3. Results

A total of 134 (out of 157) UG students participated in this study. The samples consisted of 97 males (72.4%) and 37 females (27.6%). 

### 3.1. Students’ Knowledge and Attitude towards Plg by Gender Variables

As compared to the proportion of male students, the percentage of female students responding to this survey was lower as only three batches of female dental students are currently present in this institute as compared to the five batches of male students. Table 1 presents the Mann Whitney U test results of Plg, wherein male students were found to be more disfavoring than female students for all the statements on Plg. Statistically significant results were found between the genders on statements pertaining to meaning of Plg. On statements such as whether a plagiarized paper does no wrong to science, temptation to plagiarize, or not been caught yet, and it is not so bad to plagiarize, the females participants showed more awareness by disagreeing with these statements in a way that was statistically different from the male respondents.

### 3.2. Students’ Knowledge and Attitude towards Plg by Area of Study

For the statements pertaining to the meaning of Plg, the medical students showed better awareness than the dental students with most of the responses being statistically significant. However, for the questions related to understand that this practice is wrong, the dental students had responded well as compared to the medical students as shown in Table 2.

### 3.3. Students’ Responses on Plg by Academic Years

**Figure 1:**
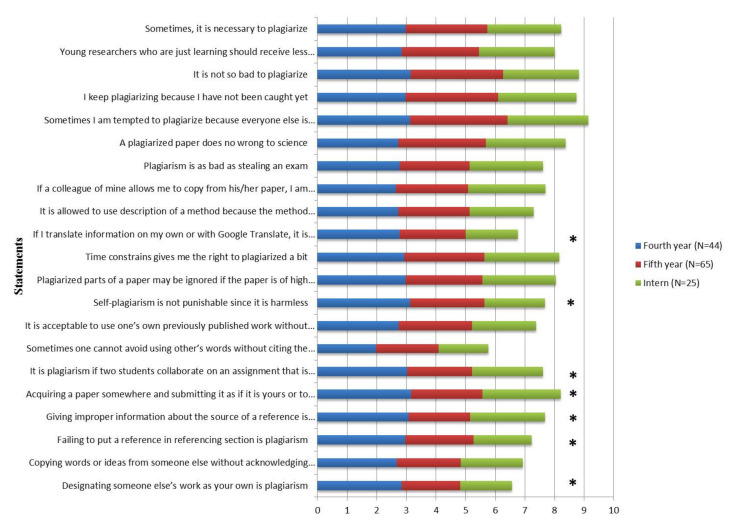
Students’ responses on plagiarism by academic years (expressed in mean). * Statistically Significant.

Out of 134 students, 32.8% were fourth year, 48.5% belonged to fifth year, and 18.7% were interns. As students climb the academic ladder, their knowledge and attitude regarding Plg tend to rise. For most of the statements, interns gave the better ratings on the meaningful and harmful effects of practicing Plg (*p* < 0.05). Figure 1 shows the academic-wise responses of study participants wherein statistically significant responses were noted for the statements, including: “designating someone else’s work as your own is plagiarism”, “failing to put a reference in referencing section is plagiarism”, “giving improper information about the source of a reference is plagiarism”, “acquiring a paper somewhere and submitting it as if it is yours or to pay somebody to do your paper is plagiarism”, “it is plagiarism if two students collaborate on an assignment that is not group work, and then submit the same assignment under two different names”, “self-plagiarism is not punishable since it is harmless”, and “if I translate information on my own or with Google translate, it is no longer the original source and so I do not need to acknowledge the source”.

### 3.4. Strategies to Prevent Plg

In spite of the increased percentage of knowledge about Plg, most of the respondents (69.4%) believed that they still need some guidance/lectures on Plg. Similarly, most of the participants agreed that Plg should be discussed at different levels from UG to postgraduate levels (59.7%), students’ workload should be decreased to enable them do more thorough research (52.2%), information about Plg should be displayed on university notice boards and website (50.7%), and the university should provide Plg detection tools and mandate students to submit their papers online (48.5%) (Table 3).

## 4. Discussion

This study attempted to assess the awareness of students regarding Plg at College of Medicine and Dentistry, Jouf University, as well as their strategies to prevent Plg. Although limited to two sources at the university, the study sheds light on the nature and extent of the deficiencies that afflicts academia and the student body in Saudi Arabia.

Hosny M and Fatima S (2014) carried out a study on the attitude of undergraduate and masters students towards cheating and Plg in Saudi Arabia and found that the level of awareness of Plg by the respondents was high, as have many other earlier studies [9,10,11,12,13,14]. On the contrary, Kattan AE et al. (2017) [8] conducted a study on physicians-in-training in Saudi Arabia and found that most of the respondents had an predisposition towards Plg along with a tendency towards reduced tolerance of Plg and a preference towards personal approval of Plg practice in society. Many authors in their respective studies have also noted a deficiency of knowledge towards plagiarism [3,7,15]. Based on the current survey research, the general attitudes of the participants were found to be disfavoring Plg as they rejected most of the statements which seemed to uphold Plg. The majority of the respondents were aware of the meaning of Plg in terms of designating someone else’s work as your own (69.4%), failure to acknowledge (60.4%), failing to put a reference (56.7%), improper referencing (53%), paying for paper writing or submitting someone else’s work as own (42.5%), and sharing the same work under two different names (53%).

In this study, male students were more aware than female students for all the statements pertaining to the meaning of Plg. However, when it came to understanding that this practice is wrong, female participants agreed more as compared to males. This finding is in accordance with a study conducted by Ahmad S and Ullah A (2015) [16] as the results revealed that the use of Plg avoidance techniques was two times higher in male as compared to female students. However, according to a study performed by Ahmed SZ et al. (2017) [7], females had better knowledge about the meaning of Plg.

Among the study participants, the reasons for plagiarizing, in descending order, were the voluntary sharing of work (51.5%), time constraints (46.3%), sometimes it is mandated (42.5%), having not been caught yet (35.1%), it is not bad to plagiarize (35.1%) and being tempted (32.1%). According to Yadav P and Kasulkar AA (2017) [3], a high proportion of students resorts to cheating because they have not been caught yet. The Saudi students in the study conducted by Razek NA (2014) [17] identified cheating and dishonesty as unacceptable in Islam, yet still reported academic dishonesty as relatively acceptable since Saudi students feel pressure to excel. Similarly, Moten AR (2014) [18] reported that Plg is fairly high among Muslim students and Muslim faculty and implied that Muslim students do not feel regret of Plg unless caught. 

In the present study, when the participants were questioned about the consequences of Plg, 50% of them answered that self-Plg is not punishable, 52.2% of the respondents considered Plg to be as bad as stealing an exam, and 43.9% of participants marked that a plagiarized paper does no wrong to science. Similarly, the majority of the respondents (46.3%) believed that young researchers should receive less punishment for Plg. Earlier studies have shown that a significant reason for plagiarizing was the absence of punishment for Plg-related offences as students believed that Plg was not a serious academic offense that should attract heavy sanctions [4,19]. As a result of the data from all three sources at the university in Saudi Arabia, Al-Jarf R (2013) [20] recommends introducing stricter punishments and guidelines to Saudi Arabian higher education. Nonetheless, many countries follow strict professional penalties for Plg ranging from verbal warnings to resubmission of the work, withdrawal of the certificates to failing semester, and suspension to expulsion [3]. It is noteworthy that there is no forgiveness as a consequence to those who plagiarize in any institute around the world. [21]

In the current study, when it came to understanding that this practice is wrong, female participants agreed more as compared to males. However, according to the study conducted by Ahmed SZ et al. (2017) [7], males agreed more when they were questioned about the consequences of Plg as compared to females.

Although earlier studies have made comparisons between faculty and students or between students pursuing different professional courses [10,14], to the best of our knowledge no research has been conducted regarding comparisons among medical and dental students, as done in the current study. Regarding the level of studies, previous studies have also revealed that that the probability of using Plg avoidance techniques decreases as the level of education decreases, and supported the assumption that younger students and less academically able students were found to plagiarize more [8,16]. Similar findings were noted in the current study.

When it came to avoiding Plg, the majority of the participants (69.4%) believed that they need some guidance/workshop regarding Plg as it would improve their writing skills. Earlier studies have recognized a lack of training in research methodology and suggested formal training in research ethics and medical writing to combat Plg [7,22,23]. Hence, seminars, workshops, and lectures should be organized specifically to address this issue. Dedicated sections on research methodology and analytical and referencing techniques should be integrated in UG curriculum to further cultivate the research environment in Saudi Arabia. The other recommendations made by the respondents in the current study were that the information about plagiarism should be displayed on university notice boards and website, and that the university should provide plagiarism detection tools and mandate students to submit their papers online. Although Jouf University has proper rules and regulations regarding Plg and also offers free facilities to all students and faculty members for Plg detection through authenticated software, many students are still unaware of it and thus the students should be made well aware of these facilities to have a favorable perception and attitude towards Plg. Several strategies have been outlined in previous studies to combat Plg including familiarizing the students at an early stage about the importance of academic integrity, providing awareness about the legal consequences of plagiarized work, and the adoption of strict policies by institutions [16,24,25,26].

### Limitations of Study

Firstly, the small sample size is the main limitation. However, as such, it has served as a pilot study in the research area. Secondly, the gender differences in this study, if any, may need to be interpreted with caution as only three batches of female dental students are currently present in this institute as compared to the five batches of male students. Thirdly, the results of current study cannot be generalized to other higher education institutions in Saudi Arabic. Lastly, a use of self-administered questionnaires may lead to information bias since agreement can be subjective. 

## 5. Conclusions

This research uncovered the key insights with strategic importance. Results from this study could be considered as an important factor in overall student success, thus proving that an institution has done its job by giving students the education/training they deserved and hence improving the competence of graduates from that institution.

To avoid Plg, proper policies should be developed by the stakeholders. Workshops, seminars, and lectures should be organized specifically to address the problems of Plg, which is particularly necessary in countries when English is not a native language.

Moreover, to promote a research environment, the revision of UG and postgraduate curriculum should also be considered, which includes research methodology, referencing, and analytical methods.

Finally, the more scientific studies are conducted, the more ways of prevention could be found to inform stakeholders, and therefore more studies are needed on this topic.

## Figures and Tables

**Table 1:** Mann Whitney U test results of plagiarism by gender variables.

Statements	Male (N = 97) Female (N = 37)	Mean ± SD	Median	*p*-Value
Designating someone else’s work as your own is plagiarism	Males	2.05 ± 1.22	2	0.00 *
	Females	2.62 ± 1.19	2	
Copying words or ideas from someone else without acknowledging is plagiarism	Males	2.16 ± 1.06	2	0.03 *
	Females	2.70 ± 1.27	2	
Failing to put a reference in referencing section is plagiarism	Males	2.32 ± 1.08	2	0.03 *
	Females	2.81 ± 1.16	2	
Giving improper information about the source of a reference is plagiarism	Males	2.44 ± 1.16	2	0.69
	Females	2.57 ± 1.28	2	
Acquiring a paper somewhere and submitting it as if it is yours or to pay somebody to do your paper is plagiarism	Males	2.55 ± 1.16	2	0.04 *
	Females	3.08 ± 1.46	2	
It is plagiarism if two students collaborate on an assignment that is not group work, and then submit the same assignment under two different names	Males	2.42 ± 1.19	2	0.24
	Females	2.70 ± 1.20	2	
Sometimes one cannot avoid using other’s words without citing the author	Males	1.89 ± 0.99	2.5	0.08
	Females	2.22 ± 0.99	2	
It is acceptable to use one’s own previously published work without providing citation in current work	Males	2.39 ± 1.25	3	0.11
	Females	2.78 ± 1.23	3	
Self-plagiarism is not punishable since it is harmless	Males	2.55 ± 1.16	3	0.21
	Females	2.81 ± 1.14	2.5	
Plagiarized parts of a paper may be ignored if the paper is of high scientific importance	Males	2.7 ± 1.20	2	0.81
	Females	2.65 ± 1.23	2	
Time constrains gives me the right to plagiarized a bit	Males	2.63 ± 1.20	2	0.13
	Females	3.03 ± 1.22	2	
If I translate information on my own or with Google Translate, it is no longer the original source and so I do not need to acknowledge the source	Males	2.23 ± 1.26	2	0.20
	Females	2.54 ± 1.29	2	
It is allowed to use description of a method because the method remains the same	Males	2.36 ± 1.06	2	0.13
	Females	2.73 ± 1.22	2	
If a colleague of mine allows me to copy from his/her paper, I am not doing anything wrong	Males	2.43 ± 1.15	3	0.13
	Females	2.81 ± 1.33	3	
Plagiarism is as bad as stealing an exam	Males	2.55 ± 1.18	3	0.56
	Females	2.43 ± 1.28	3	
A plagiarized paper does no wrong to science	Males	2.66 ± 1.20	3	0.01 *
	Females	3.29 ± 1.23	3	
Sometimes I am tempted to plagiarize because everyone else is doing it	Males	2.94 ± 1.24	3	0.00 *
	Females	3.62 ± 0.96	3	
I keep plagiarizing because I have not been caught yet	Males	2.84 ± 1.15	3	0.02 *
	Females	3.38 ± 1.30	3	
It is not so bad to plagiarize	Males	2.82 ± 1.19	1	0.02 *
	Females	3.54 ± 1.08	3	
Young researchers who are just learning should receive less punishment for plagiarism	Males	2.61 ± 1.15	1	0.34
	Females	2.81 ± 1.06	2	
Sometimes, it is necessary to plagiarize	Males	2.67 ± 1.19	2	0.07
	Females	3.05 ± 1.25	2	

Scale: 1 = strongly agree, 2 = agree, 3 = neutral, 4 = disagree, and 5 = strongly disagree. * Statistically Significant.

**Table 2:** Mann Whitney U test results of plagiarism by area of study.

Statements	Medical (N = 88) Dental (N = 46)	Mean ± SD	Median	*p*-Value
Designating someone else’s work as your own is plagiarism	Medical	1.97 ± 1.09	2	0.00 **
	Dental	2.67 ± 1.37	2	
Copying words or ideas from someone else without acknowledging is plagiarism	Medical	2.22 ± 1.15	2	<0.001 **
	Dental	2.5 ± 1.12	2	
Failing to put a reference in referencing section is plagiarism	Medical	2.39 ± 1.00	2	0.68
	Dental	2.59 ± 1.31	2	
Giving improper information about the source of a reference is plagiarism	Medical	2.32 ± 1.15	2	1
	Dental	2.78 ± 1.21	2	
Acquiring a paper somewhere and submitting it as if it is yours or to pay somebody to do your paper is plagiarism	Medical	2.63 ± 1.26	2	0.42
	Dental	2.83 ± 1.29	2	
It is plagiarism if two students collaborate on an assignment that is not group work, and then submit the same assignment under two different names	Medical	2.43 ± 1.17	2	0.52
	Dental	2.63 ± 1.27	2	
Sometimes one cannot avoid using other’s words without citing the author	Medical	1.88 ± 0.96	2	0.10
	Dental	2.19 ± 1.03	3	
It is acceptable to use one’s own previously published work without providing citation in current work	Medical	2.29 ± 1.19	2	0.01 **
	Dental	2.89 ± 1.28	2	
Self-plagiarism is not punishable since it is harmless	Medical	2.48 ± 1.16	3	0.04 **
	Dental	2.89 ± 1.11	3	
Plagiarized parts of a paper may be ignored if the paper is of high scientific importance	Medical	2.63 ± 1.21	2	0.55
	Dental	2.80 ± 1.21	2	
Time constrains gives me the right to plagiarized a bit	Medical	2.61 ± 1.23	2	0.11
	Dental	2.98 ± 1.17	2	
If I translate information on my own or with Google Translate, it is no longer the original source and so I do not need to acknowledge the source	Medical	2.03 ± 1.12	2	0.00 **
	Dental	2.85 ± 1.38	3	
It is allowed to use description of a method because the method remains the same	Medical	2.33 ± 1.10	2	0.05 **
	Dental	2.72 ± 1.12	2	
If a colleague of mine allows me to copy from his/her paper, I am not doing anything wrong	Medical	2.34 ± 1.16	3	0.01 **
	Dental	2.91 ± 1.23	3	
Plagiarism is as bad as stealing an exam	Medical	2.39 ± 1.23	3	0.11
	Dental	2.74 ± 1.15	3	
A plagiarized paper does no wrong to science	Medical	2.83 ± 1.36	3	0.79
	Dental	2.85 ± 0.98	3	
Sometimes I am tempted to plagiarize because everyone else is doing it	Medical	3.07 ± 1.33	3	0.50
	Dental	3.24 ± 0.94	3	
I keep plagiarizing because I have not been caught yet	Medical	2.92 ± 1.31	3	0.42
	Dental	3.11 ± 1.00	3	
It is not so bad to plagiarize	Medical	2.93 ± 1.30	1	0.28
	Dental	3.19 ± 0.97	1	
Young researchers who are just learning should receive less punishment for plagiarism	Medical	2.51 ± 1.17	2	0.03 **
	Dental	2.98 ± 0.99	1	
Sometimes, it is necessary to plagiarize	Medical	2.60 ± 1.26	2	0.02 **
	Dental	3.11 ± 1.09	2	

** Statistically Significant.

**Table 3:** Strategies to combat plagiarism.

**Statements**	Percentage ^+^		
	Agree	Disagree	Maybe/Not Sure
Introductory lectures on plagiarism should be given at freshmen orientation programmes	69.4	20.1	10.5
Plagiarism should be discussed at different levels from undergraduate to postgraduate levels	59.7	30.6	9.7
Lecturers should lessen students’ workload to enable them do more in-depth research	52.2	26.1	21.7
Information about plagiarism should be posted on university notice boards and website	50.7	29.9	19.4
The university should introduce plagiarism detection tools and mandate students to submit their papers online	48.5	31.4	20.1

^+^ Scale: 1 = agree, 2 = disagree, 3 = maybe/not sure.

## Data Availability

The data set used in the current study will be made available on request from Rakhi Issrani; rissrani@ju.edu.sa.

## References

[1] Akbar A. (2018). Defining plagiarism: A literature review. Ethical Ling..

[2] Masic I. (2012). Plagiarism in scientific publishing. Acta Inform. Med..

[3] Yadav P., Kasulkar A.A. (2017). Knowledge and attitude of medical regarding plagiarism. World J. Pharm. Med. Res..

[4] Ibegbulam I.J., Eze J. (2015). Knowledge, perception and attitude of Nigerian students to plagiarism: A case study. IFLA J..

[5] Singh S., Remenyi D. (2016). Plagiarism and ghostwriting: The rise in academic misconduct. S. Afr. J. Sci..

[6] Selemani A., Chawinga W.D., Dube G. (2018). Why do postgraduate students commit plagiarism? An empirical study. Int. J. Educ. Integr..

[7] Ahmed S.Z., Ahmad F., Merchant M.S., Nazir M.A. (2017). Knowledge and practice of understanding plagiarism by students. Pak. J. Public Health.

[8] Kattan A.E., Alshomer F., Alhujayri A.K., Alfaqeeh F., Alaska Y., Alshakrah K. (2017). The practice and attitude towards plagiarism among postgraduate trainees in Saudi Arabia. J. Health Spec..

[9] Hosny M., Fatima S. (2014). Attitude of students towards cheating and plagiarism: University case study. J. Appl. Sci..

[10] Oyewole O., Rasheed A.A., Ogunsina S.T. (2018). Awareness, perception and attitude towards plagiarism by distance learners in University of Ibadan, Nigeria. J. Acad. Libr. Inf. Sci..

[11] Schrimsher R.H., Northrup L.A., Alverson S.P. (2011). A survey of Samford University students regarding plagiarism and academic misconduct. Int. J. Educ. Integr..

[12] Idiegbeyan-ose J., Nkiko C., Osinulu I. Awareness and Perception of Plagiarism of Postgraduate Students in Selected Universities in Ogun State, Nigeria. Libr. Philos. Pract..

[13] Louw H. (2017). Defining plagiarism: Student and staff perceptions of a grey concept. S. Afr. J. High. Educ..

[14] Shimi S.G.M., Nagesh L., Sujatha B.K. (2014). Assessment of the attitude towards plagiarism among dental postgraduate students and faculty members in Bapuji Dental College and Hospital, Davangere—A cross sectional survey. IOSR J. Dent. Med. Sci..

[15] Shirazi B., Jafarey A.M., Moazam F. (2010). Plagiarism and the medical fraternity: A study of knowledge and attitudes. J. Pak. Med. Assoc..

[16] Ahmad S., Ullah A. (2015). Self-assessment of the use of plagiarism avoiding techniques to create ethical scholarship among research students. Int. J. Manag. Knowl. Learn..

[17] Razek N.A. (2014). Academic Integrity: A Saudi Student Perspective. Acad. Educ. Leadersh. J..

[18] Moten A.R. (2014). Academic Dishonesty and Misconduct: Curbing Plagiarism in the Muslim World. Intellect. Discourse.

[19] Ting S.H. Academic writing: Citation is troublesome and plagiarism is no big deal. Proceedings of the International Conference on Social Science Research.

[20] Al-Jarf R. Intellectual property and e-learning at Saudi universities: Problems and solutions. Proceedings of the Ninth International Conference on e-Learning and Software for Education.

[21] Lindahl J.F., Grace D. (2018). Students’ and supervisors’ knowledge and attitudes regarding plagiarism and referencing. Res. Integr. Peer Rev..

[22] Zainabu R. (2017). Plagiarism in Master of Education Studies at Selected East African Universities. Master’s Thesis.

[23] Rathore F.A., Waqas A., Zia A.M., Mavrinac M., Farooq F. (2015). Exploring the attitudes of medical faculty members and students in Pakistan towards plagiarism: A cross sectional survey. PeerJ.

[24] Harris R. (2015). Anti-Plagiarism Strategies for Research Papers. http://www.virtualsalt.com/antiplag.htm.

[25] Sarkodie-Mensah K. (1999). Plagiarism and the international students. Cathol. Libr. World.

[26] Basic Z., Kruzic I., Jerkovic I., Buljan I., Marusic A. (2019). Attitudes and knowledge about plagiarism among university students: Cross-sectional survey at the University of Split, Croatia. Sci. Eng. Ethics.

